# Specific Proteomic Identification of Collagen-Binding Proteins in *Escherichia coli* O157:H7: Characterisation of OmpA as a Potent Vaccine Antigen

**DOI:** 10.3390/cells12121634

**Published:** 2023-06-15

**Authors:** Ricardo Monteiro, Ingrid Chafsey, Nelly Caccia, Valentin Ageorges, Sabine Leroy, Didier Viala, Michel Hébraud, Valérie Livrelli, Mariagrazia Pizza, Alfredo Pezzicoli, Mickaël Desvaux

**Affiliations:** 1 INRAE, UCA, UMR0454 MEDIS, 63000 Clermont-Ferrand, France; 2 GSK, 53100 Siena, Italy; 3 Instituto de Investigação e Inovação em Saúde–i3S, Universidade do Porto, 4150-564 Porto, Portugal; 4 INRAE, Metabolism Exploration Platform, Proteomic Component (PFEMcp), 63122 Saint-Genès Champanelle, France; 5 UCA, INSERM, INRAE, M2ISH, 63000 Clermont-Ferrand, France; 6 Imperial College London, South Kensington Campus, London SW7 2AZ, UK

**Keywords:** extracellular matrix (ECM), collagen, intestinal epithelial cell, host cell-bacteria interaction, cell adhesion, cell surface protein, cell aggregation, conserved antigens, *Escherichia coli*, diarrhoeagenic and intestinal bacterial pathogen

## Abstract

*Escherichia coli* is a versatile commensal species of the animal gut that can also be a pathogen able to cause intestinal and extraintestinal infections. The plasticity of its genome has led to the evolution of pathogenic strains, which represent a threat to global health. Additionally, *E. coli* strains are major drivers of antibiotic resistance, highlighting the urgent need for new treatment and prevention measures. The antigenic and structural heterogeneity of enterohaemorrhagic *E. coli* colonisation factors has limited their use for the development of effective and cross-protective vaccines. However, the emergence of new strains that express virulence factors deriving from different *E. coli* diarrhoeagenic pathotypes suggests that a vaccine targeting conserved proteins could be a more effective approach. In this study, we conducted proteomics analysis and functional protein characterisation to identify a group of proteins potentially involved in the adhesion of *E. coli* O157:H7 to the extracellular matrix and intestinal epithelial cells. Among them, OmpA has been identified as a highly conserved and immunogenic antigen, playing a significant role in the adhesion phenotype of *E. coli* O157:H7 and in bacterial aggregation. Furthermore, antibodies raised against recombinant OmpA effectively reduced the adhesion of *E. coli* O157:H7 to intestinal epithelial cells. The present work highlights the role of OmpA as a potent antigen for the development of a vaccine against intestinal pathogenic *E. coli*.

## 1. Introduction

The prevention of *Escherichia coli* infections is a pressing concern from both public health and economic perspectives [1]. The wide range of diseases caused by *E. coli*, along with the high costs for healthcare systems, underlines the critical need for a broadly protective vaccine against pathogenic *E. coli* strains in modern society. The gravity of the issue was further highlighted in the first global report on antibiotic resistance published by the World Health Organization in April 2014 [2]. However, attempts to develop a safe and broadly protective vaccine against *E. coli* have thus far been unsuccessful, primarily due to the genetic and antigenic variability of pathogenic *E. coli* species. This variability, combined with the challenge of predicting vaccine coverage and effectiveness, has limited the testing of numerous promising pre-clinical candidates in human trials [3,4]. Enterohaemorrhagic *E. coli* (EHEC) are diarrhoeagenic *E. coli* (DEC) belonging to the shigatoxin-encoding *E. coli* (STEC) group. They can cause a spectrum of symptoms ranging from watery diarrhoea and bloody diarrhoea to haemorrhagic colitis (HC), haemolytic uremic syndrome (HUS) and other thrombotic microangiopathies (TMA) in humans, such as thrombotic thrombocytopenic purpura (TTP) [5]. Despite the severity of these infections, little progress has been made in reducing their incidence, and there are limited interventions available to mitigate food contamination and the infectious complications associated with this enteric disease [6]. While the vast majority of EHEC infections are sporadic, they can also give rise to major outbreaks worldwide [7]. EHEC infections and associated diseases are related to seven major serotypes, i.e., O157:H7, O26:H11, O45:H2, O103:H2, O111:H8, O121:H19 and O145:H28 [8]. These EHEC strains are responsible for causing severe symptoms, with *E. coli* O157:H7 being the most incriminated worldwide.

The adherence of EHEC on intestinal epithelial cells is believed to be the first step for developing these diseases. EHEC virulence extends beyond the toxin-mediated effects of shigatoxins (Stx). The initial and crucial step in EHEC pathogenesis is the adherence of the bacteria to intestinal epithelial cells [9]. Surface proteins constitute the most diverse group of molecular determinants involved in EHEC colonisation, including monomeric and multimeric surface colonisation factors (SCFs) [10]. In addition to Stx, the Type III secretion system (T3SS) is the most well-known molecular determinant of EHEC virulence, encoded by the chromosomal pathogenicity island LEE (locus for enterocyte effacement) [10,11]. The LEE also encodes effector proteins that are secreted via the T3SS, such as EspA (*E. coli* secreted protein A), EspB, EspD, the receptor for intimin (Tir) and the primary adhesion intimin (Eae), involved in the intimate attachment to epithelial responsible for A/E (attaching/effacing) lesion. While this phenotype is never observed clinically from histological samples of EHEC infections, some LEE-negative EHEC strains can still bind to host epithelial cells, suggesting the involvement of other adhesins and colonisation factors [8,12,13]. Several monomeric SCFs have been demonstrated to play a role in EHEC adhesion, such as Efa-1 (*E. coli* factor adherence 1), Iha (Iron-regulated protein A homologue adhesin) or Hes (Hemagglutinin from shigatoxin-encoding *E. coli*) [10,13]. In addition to homooligomeric SCFs like Saa (STEC autoagglutining adhesin) or Eib (*E. coli* immunoglobulin-binding protein), numerous supramolecular SCFs, particularly pili, are implicated in host adhesion of EHEC. These include LPF (long polar fimbriae), ECP (*E. coli* common pilus), F9, HCP (haemorrhagic *E. coli* pili) or SFP (sorbitol-fermenting fimbriae protein) [10,14,15,16]. The high antigenic and genetic variability of EHEC has limited the development of an effective and widely protective vaccine. Targeting only accessory antigens not present in all *E. coli* genomes may be insufficient. Current vaccine strategies based only on individual pathotypes and major virulence determinants have proven unsuccessful [17]. Therefore, broader strategies based on conserved features across all strains may be more effective.

To date, only one human vaccine against pathogenic bacteria was developed using the reverse vaccinology approach, namely the 4CMenB, also known as Bexsero, licenced and commercialised in many countries worldwide [18,19]. This vaccine, based on surface-exposed and conserved antigens identified from genomic analyses, is broadly protective against meningococcal group B disease [20,21]. In reverse vaccinology, the functional characterisation of protein antigens is a key step for the selection of the most promising candidates. The expression, surface localisation, function and immunogenicity of the antigen are key parameters to consider. Antigens playing a key role in adhesion can be ideal vaccine antigens, having the ability to induce antibodies able to impair this important step in pathogenicity. Adhesive or enzymatic factors contributing to the interaction with host cells in the course of an infection are more likely to be recognised by the immune system [22,23]. Instead of relying solely on genome-based analysis, this study aimed to identify potential adhesins as vaccine candidates in *E. coli* O157:H7 using a novel approach. Given the importance of intestinal colonisation in EHEC pathogenesis and the commonality of extracellular matrix (ECM) in intestinal tissue, we first investigated the adhesion of *E. coli* O157:H7 to relevant ECM components. As a proof-of-principle, we developed a novel proteomic strategy to recover and identify SCFs specifically binding to ECM components. Through a functional genetic approach, we assessed the capabilities of strains lacking the identified SCF to bind to intestinal ECM proteins and adhere to intestinal epithelial cells. Additionally, we evaluated the inhibitory effects of antibodies raised against the identified SCFs on *E. coli* O157:H7 adhesion. This initial functional characterisation highlights the potential of OmpA as a promising vaccine antigen and a candidate for therapeutic strategies against EHEC intestinal colonisation.

## 2. Results

### 2.1. Bacterial Culture Conditions Influence E. coli O157:H7 Adhesion to ECM Components of Intestinal Tissue

**Figure 1:**
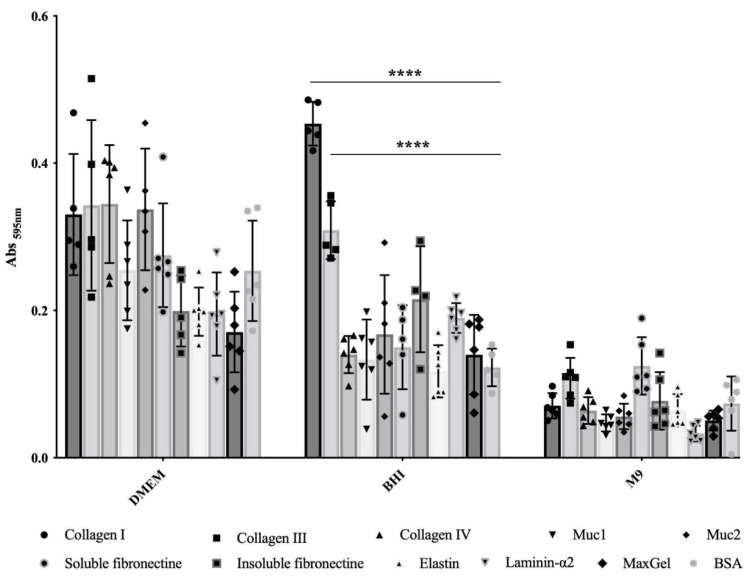
**Adhesion to immobilised ECM proteins of *E. coli* O157:H7 grown in DMEM, BHI or M9.** Specific bacterial adhesion assay to the main ECM fibrous proteins was performed at 37 °C using BSA as a control and measured by the crystal violet staining method. Data were statistically analysed following Two-way ANOVA analysis with differences considered very highly significant (*p* < 0.0001, ****).

In order to evaluate the influence of culture conditions on the adherence capability of *E. coli* O157:H7, we compared three commonly used culture media for growing EHEC. We assessed bacterial adhesion to relevant ECM components found in intestinal tissue, including FFC (fibril-forming collagen), i.e., collagen I and III, NFC (network forming collagen), i.e., collagen IV, mucin, i.e., of type 1 (muc1) and muc2, laminin, elastin, fibronectin, i.e., insoluble and soluble forms, as well as a mixture of ECM molecules (MaxGel). It became clear that adhesion ability greatly depends on the culture media (Figure 1). In the minimal chemically defined medium M9, *E. coli* O157:H7 exhibited low adhesion to ECM components, with no significant statistical differences compared to the control wells coated with BSA (Figure 1). Conversely, bacteria grown in rich chemically defined medium DMEM showed high adhesion to the different ECM proteins tested, but again with no significant statistical differences compared to the BSA control (Figure 1). These results indicate that the bacterial adhesion observed in M9 or BHI was non-specific. In contrast, when using rich and complex medium BHI, statistically significant differences were observed for bacterial adhesion to both FFC (collagens I and III), with *p*-values < 10^−4^, compared to BSA (Figure 1); these differences corresponded to a notable increase in bacterial adhesion, with a fold change of 3.7 and 2.5, respectively. However, there were no statistically significant differences observed in bacterial adhesion to the remaining tested ECM molecules compared to BSA (Figure 1). Thus, specific bacterial adhesion to collagen I and III was only observed in this particular culture condition.

### 2.2. Identification of Surface Proteins of E. coli O157:H7 Binding Specifically to Collagen I

**Figure 2:**
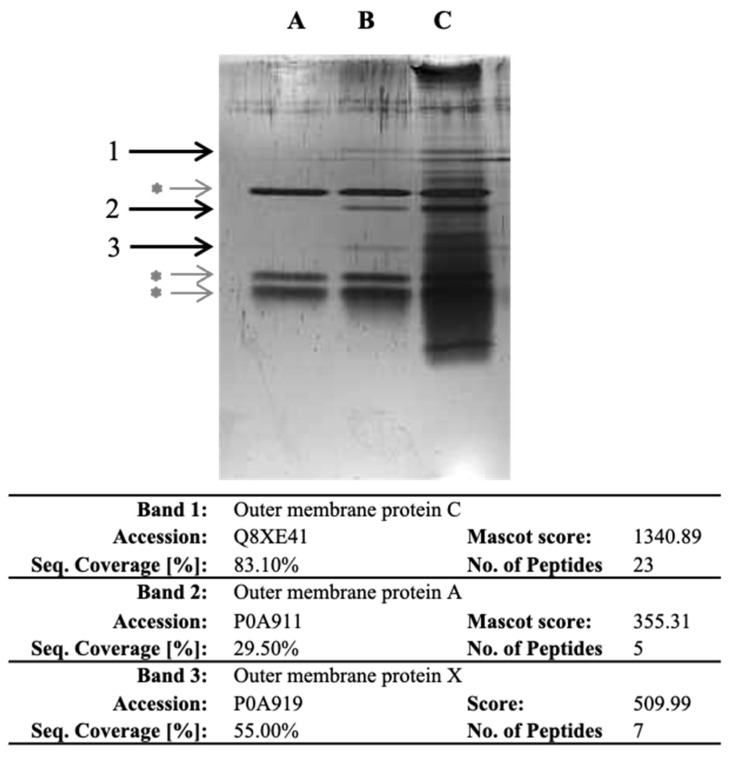
SDS-PAGE of the proteins recovered after trypsin digestion in wells with collagen and identified following LC-MS/MS analysis. After coating the OM protein fraction with collagen I, proteins were recovered and separated by SDS-PAGE. Lane A corresponds to collagen I-only. Lane B corresponds to collagen I incubated with OM protein fraction. Lane C corresponds to OM fraction-only. Bands 1, 2 and 3 are specific to collagen I-OM proteins. Grey asterisk (*) indicate bands corresponding to trypsin and auto-hydrolysed trypsin. Information on proteins identified in the specific bands is further provided in the table; detailed LC-MS/MS identification data are provided as Supplementary Material (Table S3).

As *E. coli* O157:H7 exhibited the highest specific bacterial adhesion to collagen I in BHI, our objective was to identify the bacterial proteins involved. To achieve this, we developed a novel proteomic strategy that involved isolating, recovering and identifying bacterial cell surface proteins that specifically bind to ECM proteins. Importantly, our approach did not rely on any prior assumptions or preconceptions. In the experiment, we isolated the outer membrane (OM) fraction of *E. coli* O157:H7 and exposed it to collagen I. The proteins that bound specifically to collagen were recovered through trypsin hydrolysis. Three experimental conditions were set up, namely, collagen I-only (lane A), collagen incubated with OM protein fraction (lane B) and OM protein fraction-only (lane C) (Figure 2). The recovered proteins were then separated by SDS-PAGE, and three specific bands were observed in the lane with collagen incubated with the OM fraction (Figure 2). After band excision and through LC-MS/MS analysis, OmpC, OmpA and OmpX were identified from bands 1, 2 and 3, respectively. The presence of these proteins suggested their potential involvement in the specific adhesion to collagen I.

**Figure 3:**
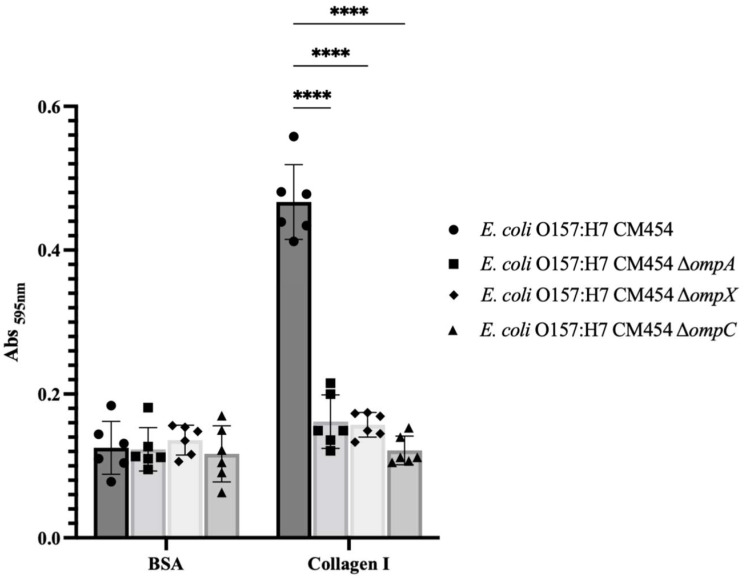
**Adhesion to immobilised collagen I of *E. coli* O157:H7 and respective deletion mutants to *ompA*, *ompX* and *ompC* genes.** Specific bacterial adhesion to collagen I of deletion mutants was compared to *E. coli* O157:H7 (black-coloured bars) and measured by the crystal violet staining method. Data were statistically analysed following ANOVA with differences considered very highly significant (*p* < 0.0001, ****). Complementation experiments are provided as Supplementary Materials (Figure S1).

To demonstrate the involvement of OmpA, OmpX and OmpC in bacterial adhesion to collagen I, functional genetic analysis was conducted, and isogenic deletion mutants were generated for each respective gene. By comparing the adhesion of these knock-out mutant strains to collagen I with *E. coli* O157:H7, it became apparent that all mutants were affected (Figure 3). Complementation experiments further confirmed the involvement of OmpA and OmpX in bacterial adhesion to collagen (Supplementary Material Figure S1) but not OmpC, for which the resulting mutant strain showed growth defect upon OmpC-induced expression.

### 2.3. Involvement of OmpA and OmpX in Bacterial Adhesion to Intestinal Epithelial Cells

**Figure 4:**
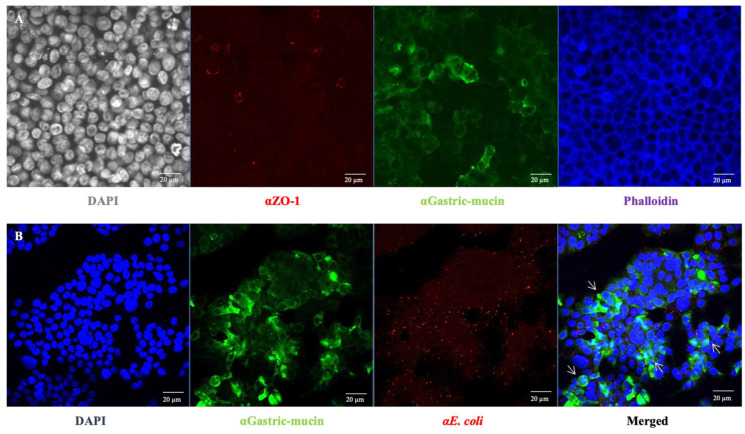
**Characterisation of the galactose-adapted HT-29 cells monolayer.** (**A**) HT-29 cells grown on a 24-well plate for 7 days were stained with specific antibodies for muc1 (αGastric-mucin) and ZO-1. The mucin is stained in green, tight junctions in red (ZO-1) and the actin skeleton in blue. DAPI (grey) staining was used to visualise cell nuclei. (**B**) The mucin is stained in green, *E. coli* O157:H7 in red and cell nuclei in blue. The white arrow indicates the co-localisation sites of bacteria and mucin.

In addition to specific bacterial adhesion to collagen I, the bacterial adhesion to intestinal epithelial cells was further investigated considering the observed high but non-specific bacterial adhesion to ECM components when *E. coli* O157:H7 was grown in DMEM (Figure 1). HT-29 cells were cultured under conditions that promoted the formation of an apical brush border, with cells differentiating into enterocytes and mucus-producing cells (goblets) [24] (Figure 4A). Seven days post-seeding, HT-29 cells formed a consistent monolayer, with approximately 40% of the cells differentiating into goblet cells that produced mucus (indicated by the green colour). At this culture stage, the infection behaviour of *E. coli* O157:H7 was evaluated by using confocal scanning laser microscopy. After one hour of incubation with HT-29 cells, *E. coli* O157:H7 (highlighted in red) had already adhered to the monolayer (Figure 4B). The co-localisation of both fluorescence signals was analysed, revealing that approximately 30% of the green signal associated with mucin overlapped with the red signal associated with bacteria. This suggested bacterial cells exhibited a stronger tendency to adhere to areas with mucin.

**Figure 5:**
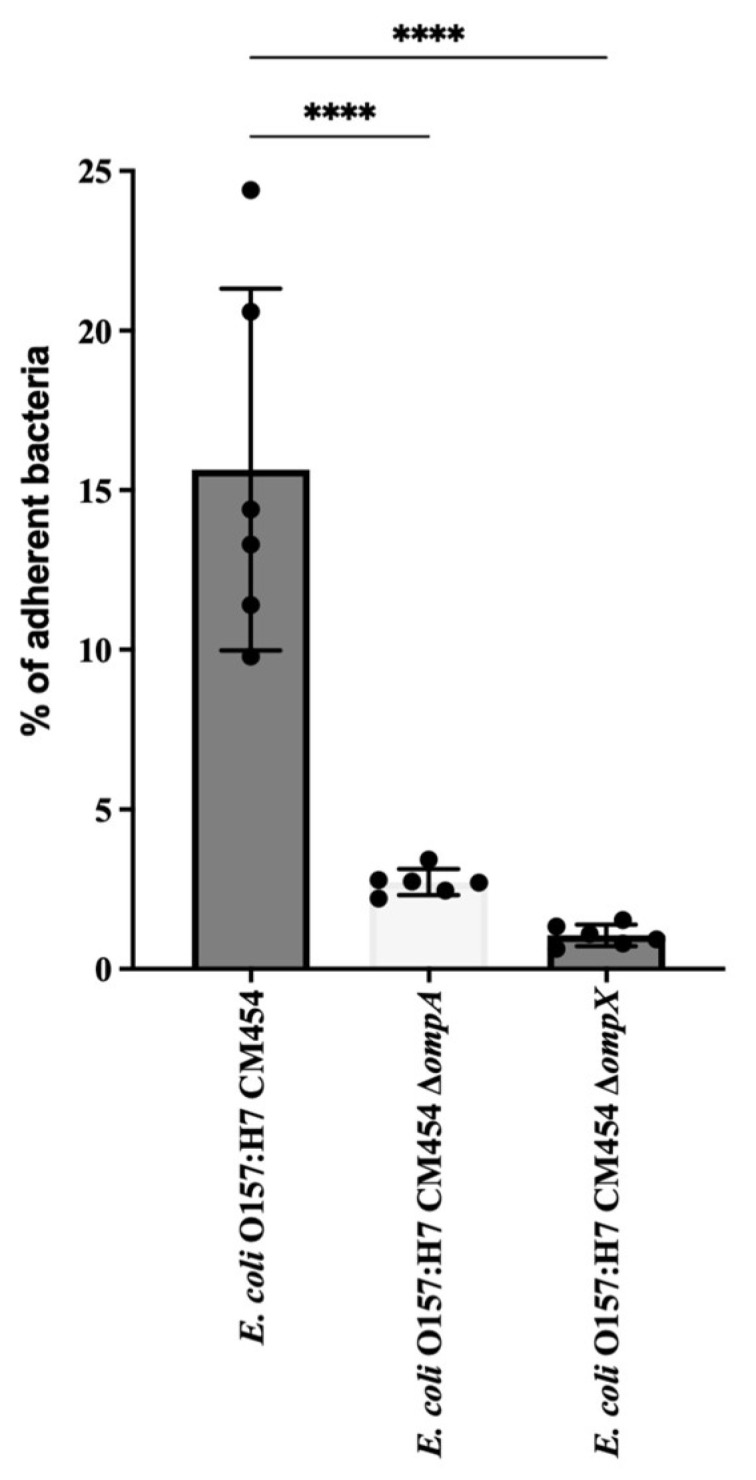
***E. coli* O157: H7 adhesion assay to HT-29 cells.** The adhesion of deletion mutant strains for *ΔompA* and *ΔompX* genes was evaluated and compared to the wild type. CFUs of adherent bacteria were counted, and mutant adhesion percentage was compared with reference *E. coli* O157:H7 (black-coloured bar). Data were statistically analysed following ANOVA with differences considered very highly significant (*p* < 0.0001, ****).

The cell adhesion of isogenic deletion mutants was compared with that of the wild-type strain of *E. coli* O157:H7 (Figure 5). The deletion of *ompA* or *ompX* genes resulted in a significant decrease in the adhesion of *E. coli* O157:H7 to HT-29 cells.

### 2.4. Involvement of OmpX and OmpA in Cell Aggregation

**Figure 6:**
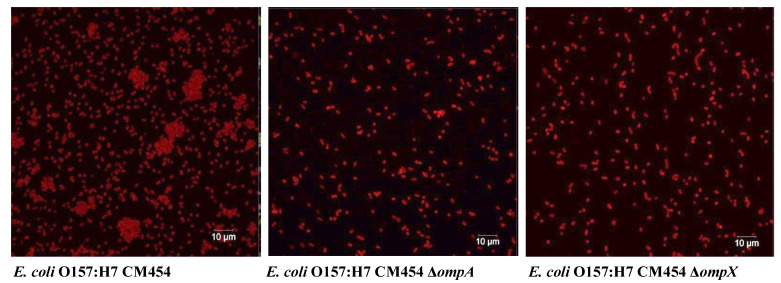
**Autoaggregation of *E. coli* O157:H7.** Bacterial cells were tagged using anti-*E. coli* antibodies with a fluorescent secondary antibody (red colour).

Since the deletion of the *ompA and ompX* genes correlated with a decrease in adhesion to HT29 monolayers, we investigated the relationship between the adhesion capability of *E. coli* O157:H7 and their localisation during cell–cell interaction. In order to visualise these cell-surface proteins, antibodies were raised against OmpA and OmpX expressing the mature proteins prior to mice immunisation; it is worth noting that the expression of OmpC in a soluble form could not be achieved due to the formation of inclusion bodies, likely attributed to hydrophobic and oligomerisation propensity of the protein. OmpA and OmpX proved to be highly immunogenic, and antibodies raised against them exhibited a clear and specific signal on the surface of *E. coli* cells under fluorescence microscopy (Figure 6).

**Figure 7:**
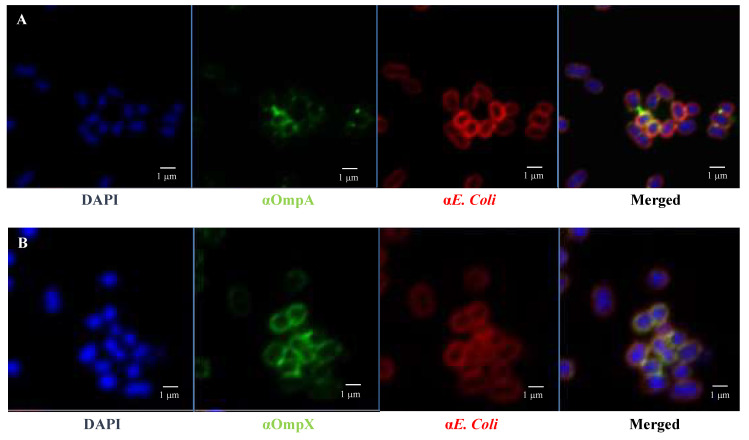
**OmpA and OmpX surface localisation in *E coli* O157:H7.** Confocal images of (**A**) OmpA (**B**) OmpX. Each protein was detected using specific antibodies raised in mice and visualised using a fluorescent secondary antibody (green). Antibodies to whole-*E. coli* bacteria and a fluorescent secondary antibody (red) and DAPI (blue) were used to visualise bacteria and chromosomal DNA, respectively.

**Figure 8:**
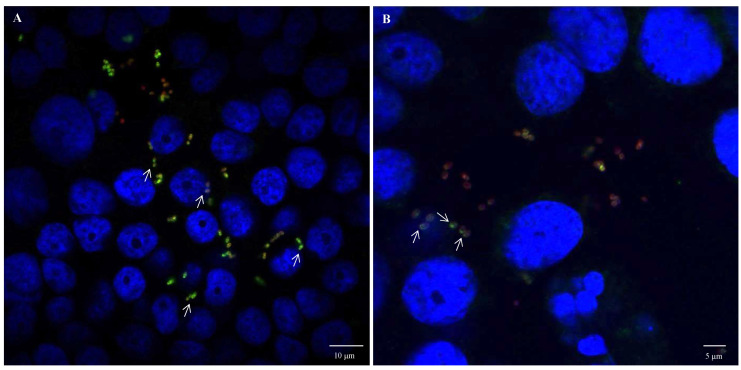
**OmpA and OmpX surface localisation during infection of HT-29 cells by *E. coli* O157:H7.** Confocal images of (**A**) OmpA and (**B**) OmpX. Each protein was detected using specific antibodies raised in mice and visualised using a fluorescent secondary antibody (green). Anti-*E. coli* antibodies with a fluorescent secondary antibody (red) and DAPI (blue) were used to visualise bacteria and chromosomal DNA, respectively. White arrows point to protein orientation to HT-29 cells.

Fluorescence microscopy images using anti-*E. coli* antibodies revealed the involvement of OmpA and OmpX in bacterial cell aggregation (Figure 6). Indeed, bacterial aggregates were absent in cultures of *E. coli* O157:H7 *ΔompA* and *ΔompX* strains. Focusing on bacterial aggregates, it appeared the fluorescence intensity of anti-OmpA and anti-OmpX antibodies (green colour) was stronger at the cell–cell interface, whereas the signal on isolated cells was either absent or significantly lower (Figure 7). In addition, during the infection of HT-29 cells, the localisation of these proteins suggested a specific orientation in the area of bacteria-cell contact (Figure 8). These results suggest OmpA and OmpX proteins would be unevenly distributed across the bacterial cell surface and tend to localise at the interface of cell interactions.

### 2.5. Anti-OmpA Antibodies Inhibit Adhesion to HT-29 Cells

**Figure 9:**
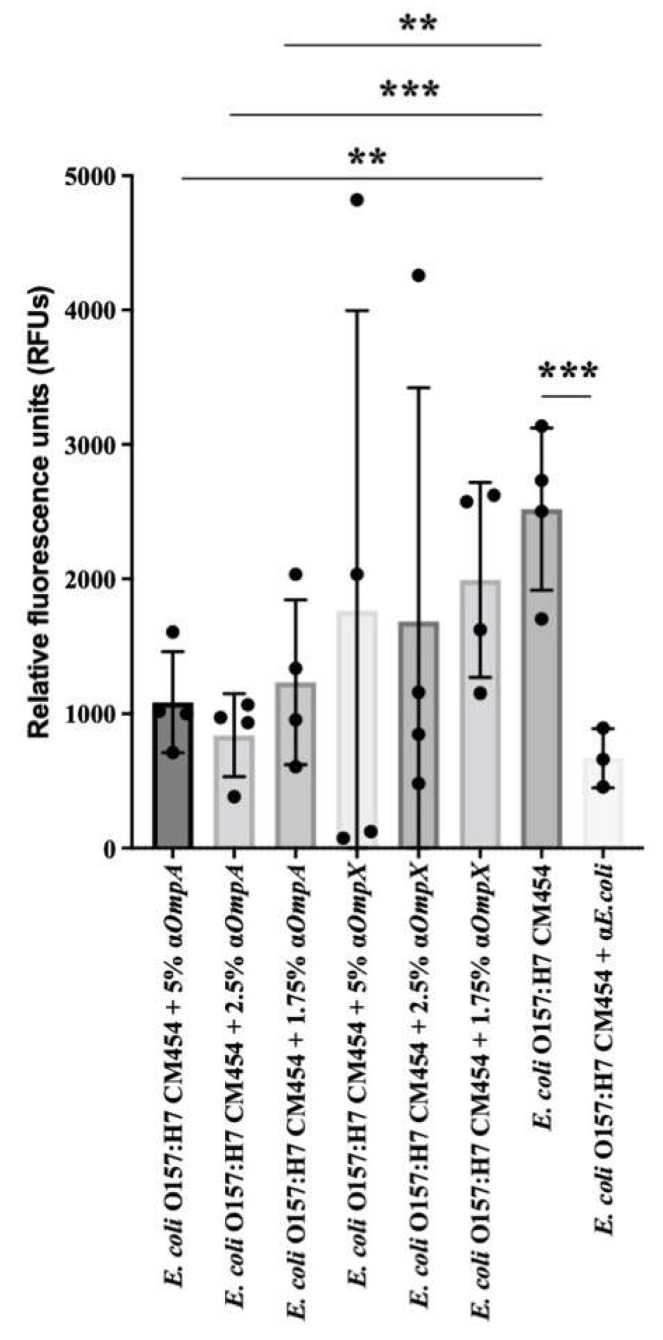
**Adhesion inhibition assay.** Effect of anti-OmpA and anti-OmpX on *E. coli* O157:H7 adhesion to HT-29 cells. The effect of 5%, 2.5% and 1.75% concentrations of anti-OmpA or anti-OmpX antibodies on bacteria-cell adhesion were evaluated and compared with *E. coli* O157:H7. Anti-*E. coli* antibodies were used as a positive control. Data were statistically analysed following ANOVA with differences considered very significant (*p* < 0.01, **), or highly significant for (*p* < 0.001, ***).

Based on the lower adhesion observed for *E. coli* O157:H7 *ΔompA* and *ΔompX* strains to intestinal epithelial cells, we further evaluated the impact of specific antibodies against OmpA and OmpX on bacterial adhesion. Pre-incubation of *E. coli* O157:H7 cells with anti-OmpX antibodies results were highly variable and the adhesion was not significantly different with respect to *E. coli* O157:H7 *wt*. However, pre-incubation of *E. coli* O157:H7 with antibodies against OmpA resulted in a statistically significant decrease in bacterial adhesion to HT-29 cells (Figure 9). Thus, the adhesion inhibition assay confirmed the efficacy of OmpA antibodies in preventing bacterial adhesion to these representative intestinal epithelial cell lines.

## 3. Discussion

Although the adhesion factors implicated in EHEC binding to intestinal epithelial cells are extensively studied, a comprehensive view of the host receptors, or the co-receptors and adhesion mechanisms remain partial. Typically, ECM proteins are localised to the epithelial basement membrane, limiting their interaction with luminal bacteria. However, interaction with enteric bacterial pathogens can occur during inflammation, anoikis, or when tight junctions are compromised [25]. In this study, we assessed the binding capability of *E. coli* O157:H7 to major intestinal ECM proteins, including mucins, collagens, laminin, elastin and fibronectin. By testing three commonly used culture media for EHEC growth, known to induce differential expression of the extracytoplasmic subproteomes [26], we observed distinct adhesion profiles to ECM proteins. High but non-specific bacterial adhesion to ECM proteins was observed in DMEM, which has been previously reported to induce bacterial adhesion due to the presence of sodium bicarbonate, a known inducer of T3aSS expression [27]. On the other hand, *E. coli* O157:H7 exhibited specific adhesion to collagen I and III in BHI, suggesting the involvement of specific surface molecular factor(s) in adhesion to FFCs. Numerous outer membrane proteins could act as MSCRAMM (microbial surface components recognising adhesive matrix molecules) proteins in DEC and be involved in bacterial adhesion to ECM proteins [9,28].

To identify potential bacterial proteins involved in specific adhesion to collagen, we developed an innovative proteomic approach. This study represents the first-time report of a strategy that allows the identification of bacterial cell-surface proteins binding specifically to ECM proteins at a cellular scale, without any prior assumptions or preconceptions. While our procedure was inspired by the work of Dreisbach et al. [29], further investigations are warranted to explore the use of high ionic strength solution alone, or in combination with tryptic digestion as an alternative or improved protocol, for releasing bacterial proteins interacting with ECM proteins.

Nevertheless, the developed protocol successfully allowed the recovery and identification of potential collagen-binding proteins, namely OmpA, OmpX and OmpC. Further characterisation revealed the involvement of OmpA and OmpX in auto-aggregation and adhesion to epithelial cells in DMEM. OmpA is known for its role in biofilm formation in *E. coli* [30], while OmpX has been implicated in *E. coli* virulence [31,32]. Both have also been reported to be involved in pathogenesis or adhesion [31,32,33,34,35]. In order to investigate bacterial adhesion to intestinal epithelial cells, HT-29 cells were differentiated into enterocytes after adaptation to galactose. It was observed that *E. coli* O157:H7 could adhere to mucus, particularly in areas of high production. Interestingly, previous studies have shown that increased mucin expression resulting from host inflammatory response inhibits *E. coli* O157:H7 adhesion to gut epithelium [36]. OmpA has been reported to bind to a less complex HeLa cell model [33] and is suggested to contribute to human meningitis by facilitating the invasion of human brain microvascular endothelial cells [34]. Regarding OmpC, attempts to induce gene expression encoding the native protein resulted in growth defect, impeding complementation, while attempts to encode the mature protein led to protein aggregates, making it challenging to generate specific antibodies. These outcomes likely arise from the hydrophobic nature of OmpC as a transmembrane porin and its ability to oligomerise, which varies in propensity and stability compared to OmpA or OmpX [37,38]. Tight and precise control of genetic expression levels is likely a prerequisite to assessing the potential contribution of OmpC to collagen and intestinal epithelial cell adhesion, but it is also quite challenging. Further comprehensive investigations are needed to determine the respective contributions and potential synergistic effects of these different OMPs to bacterial adhesion in *E. coli* O157:H7. It is important to consider the involvement of numerous other surface proteins that may be involved, including proteinaceous monomeric, oligomeric and supramolecular SCFs [10].

In addition to bacterial adhesion, these OMPs may also impact intestinal epithelial cell function. In *E. coli* O157:H7, OmpA has been implicated in the regulation of intestinal inflammation and gut barrier function [39,40,41]. In adherent-invasive *E. coli* (AIEC), a pathobiont, OmpC contributes to the virulence and infection of intestinal epithelial cells [42,43,44]. While the potential involvement of OmpX has been suggested, it has not yet been confirmed [31,45]. Therefore, in *E. coli* O157:H7, a comprehensive investigation are necessary to determine the direct or indirect effect of these OMPs on disrupting normal intestinal function, such as gut inflammation, interaction with the gut immune system and/or intestinal tissue damage, leading to diarrhoea [46].

For years, the development of vaccine strategies to prevent intestinal colonisation by DEC focused on specific enteropathotypes and their corresponding virulence factors. Examples of such strategies include toxin-based vaccines targeting shigatoxins produced by EHEC [47] or heat-labile toxins from ETEC [48]. Protein-based vaccines have also been explored, including components and effectors of the T3aSS from EPEC [26,49,50], as well as adhesins or pili from EHEC/ETEC [51,52,53]. In this study, OmpA emerged as a possible target for preventing intestinal colonisation, as antibodies against OmpA significantly reduce bacterial adhesion to intestinal epithelial cells. An ideal vaccine against DEC should include multiple antigens that induce antibodies capable of interfering with various virulence mechanisms involved in the pathogenesis of this complex pathogen. From this perspective, OmpA stands out as a potent candidate for inclusion in vaccine development against DEC.

Indeed, OmpA possesses several key attributes that make it a promising candidate for a vaccine. It is soluble, stable and highly prevalent. Recent studies have demonstrated its immunogenicity, as mice immunised with recombinant OmpA were protected against intestinal *E. coli* infection and showed cross-protection against other pathogens such as *Shigella*, *Salmonella* and *Pseudomonas* [54]. However, further investigations are still needed, considering its conservation across numerous pathogenic and commensal Gram-negative bacteria. Besides considering allelic variations, the impact of immunisation on the host microbiome should be particularly evaluated [55], as well as the efficacy of different OmpA-based formulations, in which OmpA is combined with other widely prevalent and/or pathotype-specific antigens. This makes our investigation particularly relevant and timely in the rapidly evolving field of vaccine development against pathogenic *E. coli*.

## 4. Materials and Methods

### 4.1. Cellular and Bacterial Culture Conditions

The non-toxigenic *E. coli* O157:H7 CM454 (isogenic mutant of EHEC O157:H7 EDL933) was used in this study [28,56,57]. Bacteria were cultured either in BHI (brain-heart infusion, Becton Dickinson, Germany), DMEM (Dulbecco’s modified eagle medium, Gibco) or M9. From −80 °C bacterial culture stocks, strains were plated on the appropriated agar medium and incubated overnight at 37 °C. A preculture was set up from one bacterial colony grown in the respective broth medium at 37 °C in an orbital shaker at low speed (70 rpm) until the stationary phase. For all the experiments, after 1:100 dilution, bacterial cultures were grown in the same conditions until the mid-exponential phase (~0.5 OD_600nm_).

HT-29 human colon adenocarcinoma cell line (HTB-38™, ATCC, Manassas, VA, USA) was expanded in 75 cm^2^ flasks using DMEM supplemented with 10% foetal bovine serum, 5 mM galactose and 100 μg/mL of penicillin/streptomycin until ~80% confluency and were used after third passage. For bacterial infection assays, HT-29 cells were dissociated with 0.05% trypsin-0.02% EDTA and were seeded at a density of 2.0 × 10^5^ cells/cm^2^ and grown for 7 days.

### 4.2. Generation of Isogenic Deletion Mutants and Complemented Strains

Deletion mutants for the genes coding proteins OmpA, OmpX and OmpC were generated using the Datsenko and Wanner method [58]. In short, a chloramphenicol-resistance cassette was amplified from the pKD46 plasmid using primers with homologous ends complementary to the flanking sequences of the target gene (Supplementary Materials Table S1). This PCR product was electroporated in a strain already harbouring the pKD46 plasmid expressing the λ-red genes to promote homologous recombination. Positive colonies were selected on chloramphenicol (25 µg/mL) resistance, and correct deletion of the target gene was assessed by PCR and sequencing.

Complemented strains were generated by amplifying CDS (coding DNA sequence) corresponding to the entire protein, i.e., the native OmpA, OmpX and OmpC (including signal peptide sequence), from the *E. coli* O157:H7 genomic DNA. PCR primers were designed to generate extension containing short overlapping regions with the plasmid pET15b+ (Supplementary Materials Table S1), which allowed complementary strands of the amplicons to anneal and produce hybrid vector-insert using the polymerase incomplete primer extension (PIPE) method [59]. The different plasmids pET15b-Omp-native were then transformed in each relevant knock-out strain, i.e., *E. coli* O157:H7 Δ*ompA*, or Δ*ompX* or Δ*ompC* (Supplementary Materials Table S2). Single ampicillin-resistant colonies were selected and checked for the presence of the recombinant plasmid containing the desired construct by PCR. Protein expression was induced with 0.5 mM IPTG.

### 4.3. Bacterial Adhesion Assay to ECM Proteins

The well coating of 96-well polystyrene microtiter plates (Falcon, Corning, Corning, NY, USA) with ECM proteins was performed as previously described [28,60]. The ECM proteins consisted of FFC (fibril-forming collagen), i.e., collagen I (08-115, Merck, Sigma-Aldrich, Upstate, St. Louis, MO, USA) and collagen III (CC078, Merck, Millipore, Rahway, NJ, USA), and NFC (network-forming collagen), i.e., collagen IV (C7521, Merck, Sigma-Aldrich, Merck, USA), mucin, i.e., of type 1 (muc1; M3895, Merck, Sigma-Aldrich, USA) and of type 2 (muc2; M2378, Merck, Sigma-Aldrich, USA), laminin-α2 (CC085, Merck, Millipore, USA), elastin (E1625, Merck, Sigma-Aldrich, USA), fibronectin, either insoluble (F2518, Sigma-Aldrich, Merck, Germany) or soluble (F4759, Merck, Sigma-Aldrich, USA) and MaxGel ECM (E0282, Merck, Sigma-Aldrich, USA). As a control for specific adhesion to ECM proteins, BSA (bovine serum albumin; A3803, Merck, Sigma-Aldrich, USA) was used. ECM proteins were solubilised in 0.1 M carbonate coating buffer (pH 9.6) and dispensed at a saturating concentration of 50 µg/mL to the well surface and incubated overnight at 4 °C. The wells were then washed twice with Tryptone Salt (TS) and used for bacterial adhesion assays.

Bacterial ECM adhesion assay follows the method optimised by Chagnot et al. [28]. In order to inhibit the growth and adaptation during the time of contact of bacterial cells with ECM proteins in the adhesion assay, chloramphenicol was added and mixed gently at a final concentration of 90 µg/mL to bacterial cultures. Mechanical stresses, such as vigorous shaking, vortexing and centrifugation, were avoided to preserve cell surface structures potentially involved in adhesion. *E. coli* O157:H7 cell suspension was deposited in relevant protein-coated wells of the 96-well plate using wide-bore tips. Control wells were filled with bacterial culture medium. Plates were incubated statically for 3 h at 37 °C. After incubation, non-adherent bacteria were first removed by pipetting, and wells were further washed twice with TS to remove loosely attached cells. Adherent bacteria were fixed with absolute ethanol for 20 min. The fixation solution was removed by pipetting and let to dry for 30 min. Fixed bacteria were stained with an aqueous solution of crystal violet (0.1% *w*/*v*) for 20 min. Excess of unbound crystal violet dye was washed with TS, and wells dried for 30 min. The bound dye was solubilised from stained cells using an aqueous solution of acetic acid (33% *v*/*v*) for 5 min under orbital shaking. The contents of each well were transferred to a clean 96-well plate, and absorbance was read at 595 nm using a microtiter plate reader (Tecan, Switzerland). The readings were normalised by subtracting the average absorbance from the control wells.

### 4.4. Proteomic Identification of Bacterial Surface Proteins Binding Specifically to ECM

An original proteomic strategy inspired by the work of Dreisbach et al. [29] was developed where the cell surface proteins binding specifically to ECM protein were recovered and identified. First, the outer membrane fraction of *E. coli* O157:H7 was isolated. Bacterial cells were harvested at mid-exponential phase (0.5 OD_600nm_) and pelleted by centrifugation (5000× *g* during 10 min at 4 °C) prior to resuspension in 0.1 M of Tris-HCl (pH 7.3) supplemented with 1 µL/mL of DNase. Bacterial cells were disrupted using a French press (2.5 kbar), and cellular debris was discarded by centrifugation (5000× *g* during 10 min at 4 °C). In order to enhance membrane precipitation, supernatant was diluted to a final concentration of 0.1 M of sodium carbonate and incubated for 1 h in agitation at 4 °C. Outer membrane (OM) fraction was isolated by ultracentrifugation of 120,000× *g* during 1 h, at 4 °C. Membrane pellet was washed and resuspended in 0.1 M of Tris-HCl (pH 7.3) and protein content was quantified following Bradford method [61].

From the identification of bacterial surface proteins binding specifically to collagen I, the OM fraction was added at a 1:10 ratio (*w*/*w*) (1 µg of collagen I for 10 µg of proteins in OM fraction) in the wells of microtiter plates coated with collagen I and incubated for 1 h at 37 °C. Wells were thoroughly washed twice with 0.1 M Tris-HCl (pH 7.3) to remove unbound material. Then, trypsin (V5111, Promega, France) was added at 80 ng/µL in the same buffer and incubated at 37 °C for 1 h.

As for extracytoproteomic fractions, protein samples were submitted to SDS-PAGE (sodium dodecyl sulfate–polyacrylamide gel electrophoresis) to eliminate interfering molecules (such as salts) and concentrate proteins [26]. After the addition of the same volume of Laemmli buffer (2% SDS, 25% glycerol, 5% β-mercaptoethanol, 0.005% bromophenol blue and 62.5 mM Tris-HCl, final) and heating at 95 °C for 5 min, 15 μL of protein extracts were migrated, but instead of protein concentration at the junction between stacking (4% acrylamide) and resolving (12.5% acrylamide), migration was pursued (25 mA, constant over 1h) to visualise protein bands in the resolving gel Coomassie blue staining [26]. Gel bands were excised and reduced in 100 mM ammonium bicarbonate with 45 mM dithiothreitol (DTT) at 50 °C for 45 min, and alkylated with 100 mM iodoacetamide at room temperature for 20 min in darkness. Gel bands were washed in 25 mM ammonium bicarbonate and 5% ACN for 30 min, twice in 25 mM ammonium bicarbonate and finally with 50% ACN for 30 min each. Before protein hydrolysis, gel bands were dehydrated with 100% acetonitrile and then rehydrated in 100 mM ammonium bicarbonate containing trypsin at 10 ng/µL and incubated overnight for peptide digestion. Stop of tryptic digestion and protein extraction from the gel was performed by adding 0.1% TFA in 100% ACN. Peptide mixtures were analysed by nanoflow liquid chromatography using the Ultimate 3000 RSLC (Dionex, France) with nanocapillary columns (15 cm long 75 μm internal diameter; Acclaim Pep Map RSLC, Dionex, France). The solvent gradient was increased linearly from 4% to 90% ACN in 0.5% formic acid at a flow rate of 300 nL min^−1^ for 38 min. For liquid chromatography-tandem mass spectrometry (LC-MS/MS) analysis, the elutes were electrosprayed inside an LTQ-VELOS mass spectrometer (ThermoFisher Scientific, Illkirch-Graffenstaden, France) through a nanoelectrospray ion source. Thermo Proteome Discoverer v1.3 was used for raw data file processing. The *E. coli* O157:H7 strain EDL933 database (T number T00044) was used for protein identification. For the proteomic searches, the following parameters were set up: peptide mass tolerance of 1.5 Da, fragment mass tolerance of 0.5 Da, and a maximum of two missed cleavages allowed. Variable modifications considered were carbamidomethylation (C) of cysteine and methionine oxidation (M). Proteins were considered valid when a minimum of two unique peptides originating from one protein showed statistically significant (*p* < 0.05) Mascot scores (http://www.matrixscience.com, accessed on 23 March 2023).

### 4.5. Bacterial Adhesion Assay to Intestinal Epithelial Cells

*E. coli* O157:H7 and generated isogenic mutants (*ΔompA*, *ΔompX*) were grown in DMEM and harvested at 0.5 OD_600nm_. Bacteria were pelleted and resuspended in an infection medium comprised of DMEM supplemented with 1% FBS and 5 mM galactose. The inoculum was added to HT-29 cultures previously seeded in a 24-well plate in triplicates, using a multiplicity of infection (MOI) 10:1 bacteria per cell and incubated for 1 h at 37 °C, 5% CO_2_. Subsequently, the inoculum was removed, and cells were gently rinsed three times with PBS to remove any non-adherent bacteria. Then wells are added with 1% saponin (Sigma), which lyses the cells without affecting the bacteria. Cell lysates were plated in LB, and colony-forming units (CFU) were counted. The percentage of adherent bacteria was standardised with CFU counting from inoculum pre-infection.

For adhesion inhibition assay, *E. coli* O157:H7 and generated isogenic mutants Δ*ompA* and Δ*ompX* grown in DMEM were harvested at OD_600nm_ 0.5. Bacteria were pelleted and resuspended in PBS. Bacteria were stained using Oregon green 488 (ThermoFisher Scientific, France) for 15 min at 37 °C. Bacteria were pelleted, washed in PBS and resuspended in an infection medium (DMEM supplemented with 1% FBS and 5 mM galactose). Bacterial cells were then incubated with specific sera against OmpA or OmpX in triplicates with a gradient concentration of 5%, 2.5% and 1.75% of serum for 1 h at 37 °C (inoculum). An anti-whole cell *E. coli* antibody was used as a control. HT-29 cells grown in black 96-well plates were added with inoculum and incubated at 37 °C for 1 h. Subsequently, the inoculum was removed, and the cells were gently rinsed three times with PBS to remove any non-adherent bacteria. Cells were suspended in 1% SDS and 0.1 M NaOH solution and transferred to a clean black bottom 96-well plate. Adherent bacteria fluorescence was measured at 485/535 nm using a Tecan Infinite 200PRO plate reader (Tecan, Männedorf, Switzerland).

### 4.6. Cloning, Expression and Purification of Recombinant Proteins to Generate Specific Polyclonal against Selected Omps

In order to generate specific antibodies against OmpA, OmpX and OmpC, the CDS corresponding to the mature proteins (i.e., without the signal peptide sequence) were amplified and cloned into the pET15b+ vector (Merck, Sigma-Aldrich, Novagen, USA) using the polymerase incomplete primer extension (PIPE) method [59]. Briefly, sequences coding for each protein fragment were amplified by PCR from the *E. coli* O157:H7 genomic DNA using the primers listed in Supplementary Materials Table S1. PCRs generated mixtures of incomplete extension products as short overlapping sequences were introduced at the ends of these incomplete extension mixtures by primer design, which allowed complementary strands to anneal and produce hybrid vector-insert combinations. Commercial *E. coli* HK100 cells [62] were then transformed with vector-insert hybrids. Single ampicillin-resistant colonies were selected and checked for the presence of the recombinant plasmid containing the desired construct by PCR. Plasmids from positive clones were isolated and subcloned into competent *E. coli* BL-21 (DE3) cells (Merck, Sigma-Aldrich, Novagen, USA) for protein expression. EnPresso^®^ B growth system (Merck, Sigma-Aldrich, BioSilta Oy, UK) was used for the expression of recombinant proteins according to the manufacturer’s instructions. Briefly, positive clones were cultured and diluted 1:100 in 75 mL of expression medium contained in cell culture flasks and grown overnight at 30 °C, under aeration (160 rpm). The expression medium consisted of 3 medium tablets (EnPresso^®^ B kit) dissolved in H_2_O, and it was supplemented with amylase (EnPresso^®^ B kit) and ampicillin (final concentration 100 μg/mL). On the second day, 1.5 booster tables and amylase (EnPresso^®^ B kit) were added to the bacterial culture in order to maintain cell viability and protein expression was induced by the addition of 1 mM IPTG (Merck, Sigma-Aldrich, USA). The bacteria culture was grown at 25 °C, under aeration (160 rpm). Bacteria were pelleted and resuspended in 50 mM NaH_2_PO_4_, 300 mM NaCl, 10 mM imidazole, pH 8, buffer, supplemented with protease inhibitors (cOmplete™, EDTA-free Protease Inhibitor Cocktail, Merck, Roche, Germany), bacteria cells were lysed by sonication. Cell lysate was centrifuged at 15,000× *g*, for 1 h, at 4 °C in order to collect the soluble fraction containing the expressed protein. Recombinant proteins were purified by affinity chromatography. The soluble fraction was filtered using a 0.22 µm filter, and then loaded on a 5 mL HisTrap FF Crude column (Merck, Cytiva, UK). After 20-column volume washing with 50 mM NaH_2_PO_4_, 300 mM NaCl, 30 mM imidazole, pH 8, buffer supplemented with protease inhibitors, the protein was eluted with 50 mM NaH_2_PO_4_, 300 mM NaCl, 300 mM imidazole, pH 8, buffer supplemented with protease inhibitors (Roche). For each purification, flow-through, wash and elution, fractions were separated by SDS-PAGE. Protein content was quantified using the BCA (bicinchoninic acid) assay (ThermoFisher Scientific, Pierce, Waltham, MA, USA), and purity was checked by SDS-PAGE.

Mouse serum anti-OmpA, anti-OmpX and anti-OmpC were obtained by immunising CD1 mice with two 20-μg doses of recombinant proteins adjuvanted with alum. Mouse antibodies were generated by the GSK animal facility according to standard protocols.

### 4.7. Confocal Fluorescent Microscopy

*E. coli* O157:H7 strains were harvested at OD_600nm_ 0.5, washed and resuspended in PBS. Bacteria were fixed in paraformaldehyde 2% (PFA) for 20 min on a poly-L-lysine-coated slide (Thermo Scientific). For infection of HT-29 cells, infected cells were fixed in cold methanol-acetone (50/50). After a blocking step in 10% normal goat serum (Invitrogen), slides were incubated with anti-OmpA or anti-OmpX mouse serum and then with a goat anti-mouse IgG (Jackson ImmunoResearch, West Grove, PA, USA). *E. coli* O157:H7 cells were localised using rabbit polyclonal antibodies raised against whole-cell *E. coli* and Alexa Fluor488 goat anti-rabbit IgG (ThermoFischer Scientific, Invitrogen, Waltham, MA, USA) as secondary antibodies. The samples were mounted using the Pro-Long Gold antifade reagent containing the blue-fluorescent nuclear counterstain DAPI (ThermoFischer Scientific, Pierce, USA). Images were acquired using a 64 x or 100 x oil objective (1.4 n.a.) mounted on a Zeiss LSM710 confocal microscope. Overlapping images (merge) were assembled by using the MosaiX module of AxioVision suite software (Zeiss, Périgny, France). In the pictures, the signal from OmpA and OmpX was pseudocoloured in green, while the signals from bacteria are shown in red. Co-localisation coefficients (Cc, either absolute (Cca: 0.289) or weighted (Ccw: 0.285), were estimated following image analysis using ZEN 2.3 software (Zeiss, France); values ranged from 0 to 1 (0: no pixel co-localisation, 1: all pixels co-localised). For the absolute co-localisation coefficient (Cca), the ratio of red (bacteria) pixels co-localising with the green (mucin) pixels was considered where the relative number of co-localising pixels in channel 1 (C1) or 2 (C2), respectively, were compared to the total number of pixels above the threshold. For the weighted co-localisation coefficients (Ccw), the sum of intensities of co-localising pixels in C1 or C2, respectively, were compared to the overall sum of pixel intensities above the threshold.

### 4.8. Statistical Analysis

Statistical analysis was performed using GraphPad Prism 7. Data from assays result from at least four independent experiments, i.e., four biological replicates. Error bars presented in the figures represent the standard deviation from independent experiments. The mean values from the biological replicates were compared to the mean values obtained with BSA used as a control for ECM adhesion assays (Figure 1); regarding Collagen I coating (Figure 3) and HT-29 cell adhesion (Figure 5), isogenic deletion mutants were compared with *E. coli* O157:H7 CM454; and for sera inhibition assay, different sera concentrations were compared with *E. coli* O157:H7 CM454 and anti-whole cell *E. coli* (Figure 9). Data were statistically analysed following ANOVA (analysis of variance) with differences considered very significant (*p* < 0.01, **), highly significant for (*p* < 0.001, ***), or very highly significant (*p* < 0.0001, ****).

## Data Availability

Data are provided as Supplementary Materials. Additional data that support the findings of this study can be made available upon reasonable request from the corresponding authors.

## Supplementary Materials

The following supporting information can be downloaded at: https://www.mdpi.com/article/10.3390/cells12121634/s1, Table S1: List of primers and protein sequences; Table S2: List of bacterial strains and plasmids; Table S3: LC-MS-MS identification data for OmpA, OmpX and OmpC; Figure S1: Complementation experiments of deletion mutant strains.
