# Supplementary material for: Specific Proteomic Identification of Collagen-Binding Proteins in Escherichia coli O157:H7: Characterisation of OmpA as a Potent Vaccine Antigen
Source: Cells. 2023 Jun 15;12(12):1634. doi: 10.3390/cells12121634 (PMC10297621; doi:10.3390/cells12121634)

Table S1 – List of primers and protein sequences

Name	Sequence (5' – 3')
**Deletion mutants of knock-out strains**	
OmpA_Fwd	GCACTGGCTGGTTTCGCTACCGTAGCGCAGGCCGCTCCGAAAGATAACACGTGTAG GCTGGAGCTGCTTC
OmpA_Rev	CGATCTCTACGCGACGATCCGGAGCCAGGCAGTCGATCAGTGCAGCACGCCATATG AATATCCTCCTTAGT
OmpX_Fwd	GCATGTCTTTCAGCACTGGCCGCAGTTCTGGCTTTCACCGCAGGTACTTCGTGTAG GCTGGAGCTGCTTC
OmpX_Rev	CCAACACCGGCAATCCAGGTGCCTACGTCAACGCTACGAATACGGCTCTGCATATG AATATCCTCCTTAGT
OmpC_Fwd	GTACTGTCCCTCCTGGTCCCAGCTCTGCTGGTAGCAGGCGCAGCAAACGCGTGTAG GCTGGAGCTGCTTC
OmpC_Rev	CGATCTCTACGCGACGATCCGGAGCCAGGCAGTCGATCAGTGCAGCACGCCATATG AATATCCTCCTTAGT
**Complementation of deletion mutants following expression of native recombinant proteins**	
OmpA_Fwd	CTGTACTTCCAGGGCGCTATGAAAAAGACAGCTATC
OmpA_Rev	AATTAAGTCGCGTTAAGCTTGCGGCTGAGTTACAAC
OmpX_Fwd	CTGTACTTCCAGGGCGCTCACATTTGAGGTGGTTATG
OmpX_Rev	CTGTACTTCCAGGGCGCTCACATTTGAGGTGGTTATG
OmpC_Fwd	CTGTACTTCCAGGGCGCTATGAAAGTTAAAGTACTG
OmpC_Rev	AATTAAGTCGCGTTAGAACTGGTAAACCAGACCCAG
**Cytoplasmic overexpression of mature recombinant proteins for antibody generation**	
pET15b+_OmpA_Fwd	CTGTACTTCCAGGGCGCTCCGAAAGATAACACCTGG
pET15b+_OmpA_Rev	AATTAAGTCGCGTTAAGCTTGCGGCTGAGTTACAAC
pET15b+_OmpX_Fwd	CTGTACTTCCAGGGCTCCGTAGCTGCGACTTCTAC
pET15b+_OmpX_Rev	AATTAAGTCGCGTTAGAAGCGGTAACCAACACCG
pET15b+_OmpC_Fwd	CTGTACTTCCAGGGCGGCAACAAATTAGATCTGTACGG
pET15b+_OmpC_Rev	AATTAAGTCGCGTTAGAACTGGTAAACCAGACCCAG
**Protein sequences**	
Native OmpA (Uniprot ID: P0A911)	MKKTAIAIAVALAGFATVAQAAPKDNTWYTGAKLGWSQYHDTGFINNNGPTHENQL GAGAFGGYQVNPYVGFEMGYDWLGRMPYKGSVENGAYKAQGVQLTAKLGYPITDDL DIYTRLGGMVWRADTKSNVYGKNHDTGVSPVFAGGVEYAITPEIATRLEYQWTNNI GDAHTIGTRPDNGMLSLGVSYRFGQGEAAPVVAPAPAPAPEVQTKHFTLKSDVLFN FNKATLKPEGQAALDQLYSQLSNLDPKDGSVVVLGYTDRIGSDAYNQGLSERRAQS VVDYLISKGIPADKISARGMGESNPVTGNTCDNVKQRAALIDCLAPDRRVEIEVKG IKDVVTQPQA

Native OmpX<br>(Uniprot ID: P0A919)	MKKIACLSALAAVLAFTAGTSVAATSTVTGGYAQSDAQGQMNKMGGFNLKYRYEED<br>NSPLGVIGSFTYTEKSRTASSGDYNKNQYYGITAGPAYRINDWASIYGVVGVGYGK<br>FQTTEYPTYKHDTSDYGFSYGAGLQFNPMENVALDFSYEQSRIRSVDVGTWIAGVG<br>YRF
Native OmpC<br>(Uniprot ID: Q8XE41)	MKVKVLSLLVPALLVAGAANAAEVYNKDGNKLDLYGKVDGLHYFSDDKSVDGDQTY<br>MRLGFKGETQVTDQLTGYGQWEYQIQGNSAENENNSWTRVAFAGLKFQDVGSFDYG<br>RNYGVVYDVTSWTDVLPEFGGDTYGSDNFMQQRGNGFATYRNTDFFGLVDGLNFAV<br>QYQGKNGSVSGEGMTNNGREALRQNGDGVGGSITYDYEGFGIGAAVSSSKRTDDQN<br>SPLYIGNGDRAETYTGGLKYDANNIYLAAQYTQTYNATRVGSLGWANKAQNFEAVA<br>QYQFDFGLRPSLAYLQSKGKNLGVINGRNYDDEDILKYVDVGATYYFNKNMSTYVD<br>YKINLLDDNQFTRDAGINTDNIVALGLVYQF
Mature OmpA	APKDNTWYTGAKLGWSQYHDTGFINNNGPTHENQLGAGAFGGYQVNPYVGFEMGYD<br>WLGRMPYKGSVENGAYKAQGVQLTAKLGYPITDDLDIYTRLGGMVWRADTKSNVYG<br>KNHDTGVSPVFAGGVEYAITPEIATRLEYQWTNNIGDAHTIGTRPDNGMLSLGVSY<br>RFGQGEAAPVVAPAPAPAPEVQTKHFTLKSDVLFNFNKATLKPEGQAALDQLYSQL<br>SNLDPKDGSVVVLGYTDRIGSDAYNQGLSERRAQSVVDYLISKGIPADKISARGMG<br>ESNPVTGNTCDNVKQRAALIDCLAPDRRVEIEVKGIKDVVTQPQA
Mature OmpX	SVAATSTVTGGYAQSDAQGQMNKMGGFNLKYRYEEDNSPLGVIGSFTYTEKSRTAS<br>SGDYNKNQYYGITAGPAYRINDWASIYGVVGVGYGKFQTTEYPTYKHDTSDYGFSY<br>GAGLQFNPMENVALDFSYEQSRIRSVDVGTWIAGVGYRF
Mature OmpC	GNKLDLYGKVDGLHYFSDDKSVDGDQTYMRLGFKGETQVTDQLTGYGQWEYQIQGN<br>SAENENNSWTRVAFAGLKFQDVGSFDYGRNYGVVYDVTSWTDVLPEFGGDTYGSDN<br>FMQQRGNGFATYRNTDFFGLVDGLNFAVQYQGKNGSVSGEGMTNNGREALRQNGDG<br>VGGSITYDYEGFGIGAAVSSSKRTDDQNSPLYIGNGDRAETYTGGLKYDANNIYLA<br>AQYTQTYNATRVGSLGWANKAQNFEAVAQYQFDFGLRPSLAYLQSKGKNLGVINGR<br>NYDDEDILKYVDVGATYYFNKNMSTYVDYKINLLDDNQFTRDAGINTDNIVALGLV<br>YQF

Table S2 – List of bacterial strains and plasmids

Plasmid/Strain	Description	Source
Strains		
E. coli O157:H7 CM454	Isogenic mutant of EHEC O157:H7 EDL933.	[37]
E. coli O157:H7 CM454 Δ*ompA*	*E. coli* O157:H7 CM454 lacking the gene *ompA*	This study
E. coli O157:H7 CM454 Δ*ompC*	*E. coli* O157:H7 CM454 lacking the gene *ompC*	This study
E. coli O157:H7 CM454 Δ*ompX*	*E. coli* O157:H7 CM454 lacking the gene *ompX*	This study
Escherichia coli HK100	Strain production host	[45]
BL-21 (DE3)	Competent Cells for Protein Expression; F^- *ompT* *hsdS*$_B$ ($r_B^- m_B^-$) gal dcm (DE3)	Novagen
Plasmids		
pKD46	Vector for RED-recombineering – site-targeted modification of any locus in *E. coli*.	Addgene
pET15b+	Vector system for cloning and expression of recombinant proteins in *E. coli*.	Novagen
pET15b-OmpA-native	Vector for inducible expression of native OmpA	This study
pET15b-OmpX-native	Vector for inducible expression of native OmpX	This study
pET15b-OmpC-native	Vector for inducible expression of native OmpC	This study
pET15b-OmpA-mature	Vector for inducible expression of mature OmpA	This study
pET15b-OmpX-mature	Vector for inducible expression of mature OmpX	This study
pET15b-OmpC-mature	Vector for inducible expression of mature OmpC	This study

Table S3 – LC-MS/MS identification data for OmpC, OmpA and OmpX

Internal ID: E-PP-BaDiAl-5781(B2)_RB2 / E-PP-BaDiAl-5781(B2)_RB2_01_6231.d

Band 1:	Outer membrane protein C		
Search Type:	Combined MS/MS		
Accession:	Q8XE41	Mascot score:	1340.89
Seq. Coverage:	83.10%	No. of Peptides:	23
MW [kDa]:	40.50	pI:	4.55

Sequence matches:
MKVKVLSLLVPALLVAGAANA AEVYNKDGNKLDLYGKVDGLHYFSDDKSVDGDQTYMRL
GFKGETQVTDQLTGYGQWEYQIQGNSAENENNSWTRVAFAGLKFQDVGSFDYGRNYGVVY
DVTSWTDVLPEFGGDTYGSDNFMQQRGNGFATYRNTDFFGLVDGLNFAVQYQGKNGSVSG
EGMTNNGREALRQNGDGVGGSITYDYEGFGIGAAVSSSKRTDDQNSPLYIGNGDRAETYT
GGLKYDANNIYLAAQYTQTYNATRVGSLGWANKAQNFEAVAQYQFDFGLRPSLAYLQSKG
KNLGVINGRNYDDEDILKYVDVGATYYFNKNMSTYVDYKINLLDDNQFTRDAGINTDNIV
ALGLVYQF

Cmpd.	No. of Cmpds.	m/z meas.	Δ m/z [ppm]	z	Rt [min]	Score	P	Range	Sequence	Modification (oxidation)
193	1	648.2983	-0.66	2	19.0	19.4	0	38-48	K.VDGLHYFSDDK.S	
65	1	586.2563	0.38	2	14.3	55.1	0	49-58	K.SVDGDQTYMR.L	
28	1	594.2536	0.20	2	11.7	40.9	0	49-58	K.SVDGDQTYMR.L	9
1039	3	1268.5659	0.72	3	31.2	161.9	0	63-95	K.GETQVTDQLTGYGQWEYQIQGNSAENENNSWTR.V	
277	3	645.7915	0.53	2	21.7	70.3	0	103-113	K.FQDVGSFDYGR.N	
2244	2	915.9123	1.41	4	41.5	93.8	0	114-145	R.NYGVVYDVTSWTDVLPEFGGDTYGSDNFMQQR.G	
2084	2	1226.2127	1.69	3	40.4	82.0	0	114-145	R.NYGVVYDVTSWTDVLPEFGGDTYGSDNFMQQR.G	29
76	1	443.2146	0.63	2	14.9	20.1	0	146-153	R.GNGFATYR.N	
1933	2	1117.0516	1.55	2	39.3	86.7	0	154-173	R.NTDFFGLVDGLNFAVQYQGK.N	
22	1	690.7981	718.66	2	11.8	30.7	0	174-187	K.NGSVSGEGMTNNGR.E	
955	2	1318.6111	1.43	2	30.6	90.9	0	192-218	R.QNGDGVGGSITYDYEGFGIGAAVSSSK.R	
126	1	607.6236	2.25	3	17.3	35.0	1	219-234	K.RTDDQNSPLYIGNGDR.A	
202	1	832.8805	1.56	2	19.4	57.9	0	220-234	R.TDDQNSPLYIGNGDR.A	
58	1	470.2425	-0.58	2	14.0	60.0	0	235-243	R.AETYTGGLK.Y	
748	4	785.3739	1.02	3	28.7	87.3	0	244-263	K.YDANNIYLAAQYTQTYNATR.V	
149	1	466.2535	0.23	2	18.0	54.0	0	264-272	R.VGSLGWANK.A	
1582	2	997.8401	1.90	3	35.9	111.4	0	273-298	K.AQNFEAVAQYQFDFGLRPSLAYLQSK.G	
199	1	562.7592	0.47	2	19.4	40.2	0	309-317	R.NYDDEDILK.Y	
426	2	720.3460	0.32	2	24.8	67.2	0	318-329	K.YVDVGATYYFNK.N	
77	1	568.7507	1.08	2	14.6	46.1	0	330-338	K.NMSTYVDYK.I	2
153	1	560.7532	1.04	2	18.2	45.6	0	330-338	K.NMSTYVDYK.I	

553	4	674.8464	0.04	2	26.3	72.6	0	339-349	K.INLLDDNQFTR.D	
2563	3	961.9982	1.73	2	44.4	80.5	0	350-367	R.DAGINTDNIVALGLVYQF.-	

Internal ID: E-PP-BaDiAl-5783(B4) / E-PP-BaDiAl-5783(B4)_RB4_01_6233.d

Band 2:	Outer membrane protein A		
Search Type:	Combined MS/MS		
Accession:	P0A911	Mascot score:	355.31
Seq. Coverage:	29.50%	No. of Peptides:	5
MW [kDa]:	37.20	pI:	5.98

Sequence matches: *MKKTAIAIAVALAGFATVAQA* APKDNTWYTGAKLGWSQYHDTGFINNNGPTHENQLGAG
AFGGYQVNPYVGFEMGYDWLGRMPYKGSVENGAYKAQGVQLTAKLGYPITDDLDIYTRLG
GMVWRADTKSNVYGKNHDTGVSPVFAGGVEYAITPEIATRLEYQWTNNIGDAHTIGTRPD
NGMLSLGVSYRFGQGEAAPVVAPAPAPAPEVQTKHFTLKSDVLFNFNKATLKPEGQAALD
QLYSQLSNLDPKDGSVVVLGYTDRIGSDAYNQGLSERRAQSVVDYLISKGIPADKISARG
MGESNPVTGNTCDNVKQRAALIDCLAPDRRVEIEVKGIKDVVTQPQA

Cmpd.	No. of Cmpds.	m/z meas.	Δ m/z [ppm]	z	Rt [min]	Score	P	Range	Sequence	Modification (oxidation)
84	1	528.2427	-1.12	2	17.3	36.3	0	25-33	K.DNTWYTGAK.L	
1016	3	827.9200	0.26	2	31.5	94.2	0	104-117	K.LGYPITDDLDIYTR.L	
863	2	867.7703	0.76	3	30.4	82.3	0	135-159	K.NHDTGVSPVFAGGVEYAITPEIATR.L	
659	1	874.4250	0.65	4	28.3	59.6	0	160-190	R.LEYQWTNNIGDAHTIGTRPDNGMLSLGVSYR.F	23
328	2	744.7268	-0.03	3	23.7	82.8	0	191-213	R.FGQGEAAPVVAPAPAPAPEVQTK.H	

Internal ID: E-PP-BaDiAl-5784(B5)_RB5 / E-PP-BaDiAl-5784(B5)_RB5_01_6234.d

Band 3:	Outer membrane protein X		
Search Type:	Combined MS/MS		
Accession:	P0A919	Mascot score:	509.99
Seq. Coverage:	55.00%	No. of Peptides:	7
MW [kDa]:	18.60	pI:	7.38

Sequence matches: *MKKIACLSALAAVLAFTAGTSVA* ATSTVTGGYAQSDAQGQMNKMGGFNLKYRYEEDNSP
LGVIGSFTYTEKSRTASSGDYNKNQYYGITAGPAYRINDWASIYGVVGVGYGKFQTTEYP
TYKHDTSDYGFSYGAGLQFNPMENVALDFSYEQSRIRSVDVGTWIAGVGYRF

Cmpd.	No. of Cmpds.	m/z meas.	Δ m/z [ppm]	z	Rt [min]	Score	P	Range	Sequence	Modification (oxidation)
816	2	823.3934	1.66	3	31.3	77.5	1	51-71	K.YRYEEDNSPLGVIGSFTYTEK.S	

1029	2	1075.0042	1.86	2	33.3	107.0	0	53-71	R.YEEDNSPLGVIGSFTYTEK.S	
1030	1	899.4628	0.76	2	33.4	79.7	0	96-112	R.INDWASIYGVVGVGYGK.F	
119	1	639.3059	-0.20	2	18.6	38.3	0	113-122	K.FQTTEYPTYK.H	
1575	1	1215.8677	0.62	3	37.9	80.8	0	123-154	K.HDTSDYGFSYGAGLQFNPMENVALDFSYEQSR.I	
1168	2	1221.1998	1.03	3	34.5	114.7	0	123-154	K.HDTSDYGFSYGAGLQFNPMENVALDFSYEQSR.I	19
711	1	740.3840	1.13	2	30.5	92.8	0	157-170	R.SVDVGTWIAGVGYR.F	

Figure S1 – Complementation experiments of deletion mutant strains in *ompA* and *ompX* genes

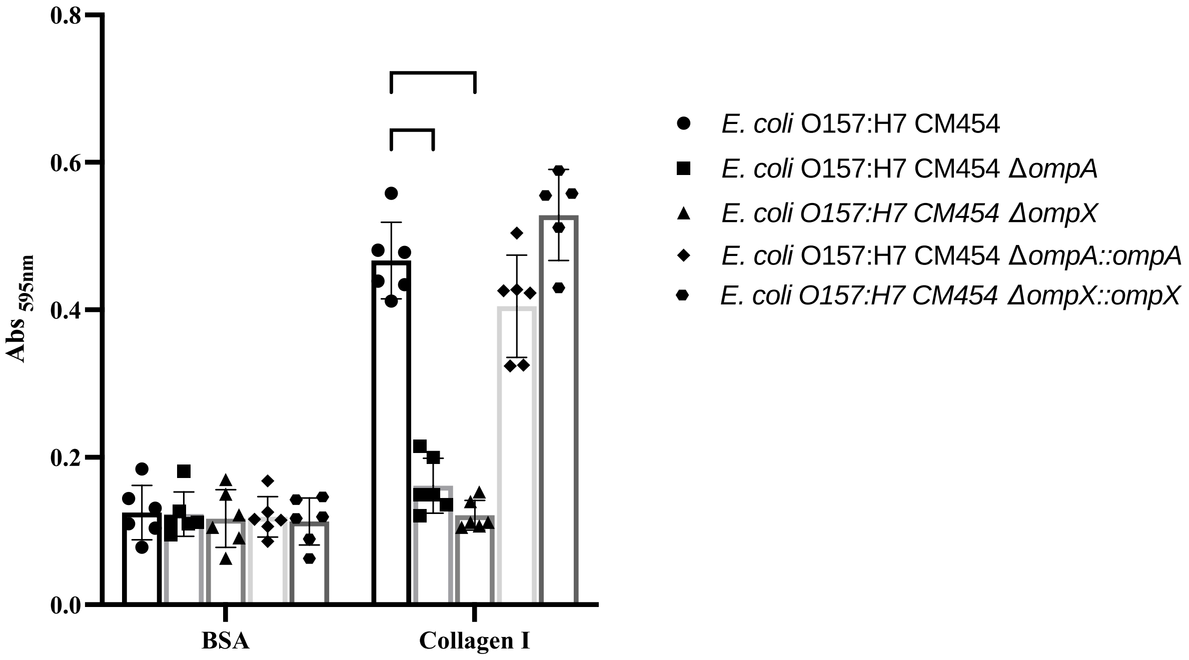

Supplement: Supplementary file 1 [file cells-12-01634-s001.zip › cells-2333382-supplementary.pdf]
